# Bacterial profile and antimicrobial susceptibility pattern in septicemia suspected patients attending Gondar University Hospital, Northwest Ethiopia

**DOI:** 10.1186/1756-0500-6-283

**Published:** 2013-07-22

**Authors:** Mulat Dagnew, Gizachew Yismaw, Mucheye Gizachew, Alemayehu Gadisa, Tigist Abebe, Tinebeb Tadesse, Agersew Alemu, Biniam Mathewos

**Affiliations:** 1 Department of Medical Microbiology, College of Medicine and Health Sciences, Gondar teaching Hospital, Gondar University, Gondar, Ethiopia; 2 Department of Parasitology, College of Medicine and Health Sciences, Gondar teaching Hospital, Gondar University, Gondar, Ethiopia; 3 Department of Immunology and Molecular Biology, College of Medicine and Health Sciences, Gondar teaching Hospital, Gondar University, Gondar, Ethiopia; 4 School of Biomedical and Laboratory Sciences, College of Medicine and Health Sciences, Gondar teaching Hospital, Gondar University, Gondar, Ethiopia

**Keywords:** Blood stream infection, Bacterial isolates, Antimicrobial susceptibility pattern

## Abstract

**Background:**

Bacterial blood stream infection constitutes a significant public health problem and it is an important cause of morbidity and mortality in hospitalized patients. The aim of this study was to assess the prevalence of bacterial isolates from septicemia suspected patients and their antimicrobial susceptibility pattern in Gondar University Hospital.

**Methods:**

This laboratory based retrospective study of 390 blood culture and susceptibility tests was conducted in Bacteriology Laboratory of the University of Gondar Teaching Hospital. The samples were collected and processed following standard microbiological techniques as part of the routine clinical management of the patient. Antibiotic susceptibility testing was done on pure culture isolates employing disc-diffusion method for the commonly used antibiotics. The data were analyzed by using SPSS version 16 and the results were summarized by using tables and graphs.

**Results:**

Out of 390 blood culture results, 71 (18.2%) were culture positive. The predominant bacteria isolated from blood culture were Coagulase negative staphylococci 30 (42.3%), followed by *S. aureus* 17 (23.9%) and *Klebiesella* spp 9 (12.9%), *E. coli* 5 (7.0%), *Pseudomonas aeroginosa* 4 (5.6%) and *Salmonella* spp. 3 (4.2%). The gram positive and gram negative bacteria constituted 49 (69%) and 22 (31%) of the culture isolates; respectively. The isolates showed high rates of resistance to most antibiotics tested. The range of resistance for Gram positive and Gram negative were from 23.5% – 58.8%, and 20%– 100% respectively.

**Conclusions:**

In the present study most of the pathogens isolated from blood culture showed high rate of resistance to most commonly used antibiotics used to treat bacterial infections. Therefore, rational use of antibiotics should be practiced.

## Background

Blood stream infection (BSI) remains one of the most important causes of morbidity and mortality throughout the world. Approximately 200,000 cases of bacteremia occur annually with mortality rates ranging from 20-50% worldwide [1]. Blood stream infection (BSI) accounts for 10-20% of all nosocomial infections and is the eighth leading cause of mortality, in the United States some 17% of result in death [2]. In sub Saharan countries including Ethiopia septicemia is an important cause of illness and death in children, the mortality rate approaches 53% which makes it a significant health problem in developing countries [3].

In many studies a wide range of bacteria has been described in febrile patients including gram negative bacteria such as *Escherichia coli, Pseudomonas aeroginosa, Klebsiella species, Neisseria meningitidis, Haemophilus influenzae*, and gram positive such as Coagulase negative staphylococci (CONS), *Staphylococcus aureus*, *Streptococcus pneumoniae*, *Streptococcus pyogenes, Streptococcus agalactiae*, and *Enterococcus faecium*[4-8]. The diagnosis of these infections can be confirmed by blood culture, which is routinely available in few hospitals in developing countries [9].

Bacterial pathogens isolated from BSI are a leading cause of significant patient morbidity and mortality. The impact of specific etiologic agents on BSI patient outcome are tremendous; BSI increases the mortality rate, prolongs patient stay in an intensive care unit and in the hospital, and leads to increased health care costs [10,11].

The timely and appropriate use of antibiotics is currently the only way to treat bacteremia. However, many bacterial pathogens have become resistant to antibiotic regimens and become a serious public health concern with economic and social implications throughout the world. Antibiotics resistance is a growing problem in developing countries such as Ethiopia. In Ethiopia the unregulated over-the-counter sale of these antimicrobials, mainly for self-treatment of suspected infection in humans, and to a lesser extent for use in animals without prescription, would inevitably lead to emergence and rapid dissemination of resistance [12]. Many studies have found that inadequate empirical therapy of bacteraemic infections is associated with adverse outcomes, including increased mortality and increased drug resistance emergence [13-15].

However, there are only a few studies from Ethiopia, which have studied the organisms involved in BSI and their susceptibility pattern. We therefore conducted this study to determine the common bacterial agents associated with BSI and their antimicrobial susceptibility patterns in Gondar tertiary teaching Hospital in Northwest Ethiopia.

## Methods

### Study design and area

A retrospective cross sectional study was conducted based on review of records of 390 patients for whom blood culture were routinely processed in department of Microbiology Laboratory of University of Gondar Hospital from September 2006 to January 2012.

### Data collection and laboratory procedure

Data on sociodemographic variables such as age, sex, blood culture results, antibiotic susceptibility pattern and sender were collected manually by using a pre-prepared data abstraction format from the department of clinical bacteriology from the registration book on which laboratory findings after investigation of patient`s blood are recorded.

Two blood samples were collected aseptically from patients for routine blood culture before taking any antibiotic treatment in the hospital. The vein puncture site was disinfected with 70% alcohol and 2% tincture of iodine before collecting approximately 10 ml of blood for culture. For blood culture 5 ml of blood was inoculated into 50 ml of Brain Heart Infusion broth (Oxoid UK). Subculture and identification of isolates were as described previously [5,7,12,16]. Two aerobic blood culture bottles were used for each patient and growth in both bottles was considered positive. Finally biochemical test was undertaken to classify bacteria at species level such as catalase, coagulase, novobiocin and optochin disk for gram positive and triple sugar iron, indole, citrate, urea, Lysine decarboxylase (LDC) and motility were for Gram negative bacteria following standard procedures [16]. Records showed that susceptibility testing was performed on Muller Hinton agar (Oxoid, Hampshire, UK) using agar disc diffusion method as described by the National Committee for Clinical Laboratory Standards [16]. The antimicrobials for disc diffusion testing was obtained from Oxoid in the following concentrations: ampicillin (AMP) (10 μg), amoxicillin (AMC) (30 μg), ceftriaxone (CRO) (30 μg), chloramphenicol (C) (30 μg), ciprofloxacin (CIP) (5 μg), erythromycin (E) (15 μg), gentamycin (CN) (10 μg), penicillin (P) (10 IU), trimethoprim-sulphamethoxazole (SXT) (25 μg) and tetracycline (TTC) (30 μg). The resistance and susceptibility were interpreted according to the National Committee for Clinical Laboratory Standards [16]. *Escherichia coli* (ATCC 25922), *Staphylococcus aureus* (ATCC 25923) and *Pseudomonas aeroginosa* (ATCC 27853) were used as reference strains for culture and susceptibility testing.

### Data analysis

Statistical analysis was done using SPSS version 16.00 software. The chi-square test was employed to assess the association between variables. A p-value of less than 0.05 was considered as statistical significance.

### Ethical consideration

This study was approved by Institutional Review Board (IRB) of University of Gondar. Official permission was obtained from the study site.

## Results

### Sociodemographic characterstics

**Figure 1:**
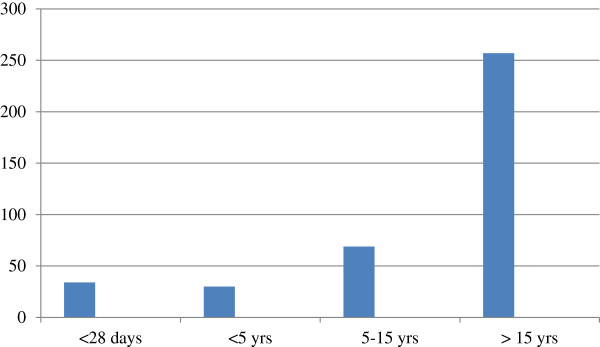
Age distribution of septicemia suspected patients at Gondar University Hospital from September 1, 2006-January 1, 2012.

Three hundred ninety of bacteremia suspected patient’s blood culture were processed routinely from September 1, 2006-January 1, 2012. Of these patients 212 (54.4%) were females and 178 (45.6%) were males. The median age of patients were 31.5 with age range 1 day- 62 years. The majority of patients 154 (39.5%) were greater than fifteen years age (Figure 1).

**Figure 2:**
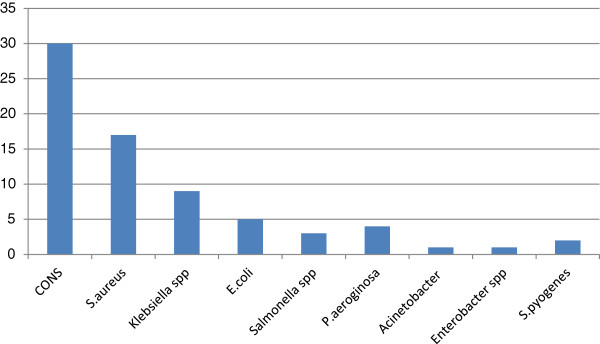
Frequency and types of bacterial isolates from septicemia suspected patients at Gondar University Hospital from September 1, 2006-January 1, 2012.

The overall prevalence of bacteria isolated from blood culture of bacteremia suspected patients were 71 (18.2%). Forty one of the culture positive were from females and 30 were from males. All infections were due to single organism. The predominant bacteria isolated from blood culture were Coagulase negative *Staphylococcus* (*CONS*) 30 (42.3%), followed by *S. aureus* 17 (23.9%) and *Klebiesella* spp. 9 (12.7%). The Gram positive and Gram negative bacteria constituted 49 (69%) and 22 (31%) of the culture isolates; respectively (Figure 2).

In the present study the predominant bacteria by age class were CONS 7 (20.6%), followed by S.aureus 2 (5.9) in neonate age (Table 1).

**Table 1:** Frequency of bacterial isolates by age class from septicemia suspected patients at Gondar University Hospital from September 1, 2006-January 1, 2012

	**Type of organism isolated n(%)**									
**Age class**	**CONS**	***S. aureus***	***E. coli***	***Klebsiella*spp.**	***Salmonella*spp.**	***P. aeroginosa***	***S. pyogen***	***Entrobacter*spp.**	***Acentob-acter*spp.**	
<28 day	7(20.6)	2(5.9)	1(2.9)	1(2.9)	1(2.9)	0	0	0		0
<5 yr	4(13.3)	4(13.3)	1(3.3)	0	0	0	0	0		0
5-15 yr	9(13)	3(4.3)	1(1.4)	3(4.3)	1(1.4)	1(1.4)	0	0		0
>15 yr	10(3.9)	8(3.1)	2(0.7)	5(1.9)	1(0.4)	3(1.2)	1(0.4)	1(0.4)	1	(0.4)

In our study, most of the sepsis patients were females 41 (57.7%), however, there was no association between sex of patient and BSI (P = 0.526). The spectrum of BSI varies with the age of patients. Thirty five point three percent of BSI was found in neonates which shares the highest proportion of sepsis patients. There was a significant association between age of patient and BSI (P = 0.0004 (Table 2). In this study 69 (97%) of bacteria were isolated from hospitalized patients while the remaining 2 (3%) were from those who attended outpatient department; however, there was no significant association between being out patient or inpatient with blood culture result (P = 0.482) (Table 2).

**Table 2:** Sociodemographic characterstics of septicemia suspected patients in relation to bacterial isolates at Gondar University Hospital since September 1, 2006- January 1, 2012

**Variable**	**Culture result**		**Association**
**Sex of patient**	**Positive n(%)**	**Negative n(%)**	**P value and*x***^**2**^
Female	41(19.3)	171(80.7)	*X*^2^ = 0.40
Male	30(16.9)	148(83.1)	P = 0.526
Total	71	319	
Age in years <28 day	12(35.3)	22(64.7)	
<5	9(30)	21(70)	*X*^2^ = 18.06
5-15	18(26.1)	51(63.9)	P = 0.0004
>15	32(12.5)	225(87.5)	
Total	71	319	
Sender			
In patient (ward)	69(18.5)	304(81.5)	*X*^2^ = 0.495
OPD	2(11.8)	15(88.2)	P = 0.482
Total	71	319	

OPD= Outpatient department.

### Antibiotic susceptibility patterns

Antimicrobial resistance levels for the Gram-negative organisms, causing blood stream infections were ranging from 20 to 100%. *Klebsiella* spp were resistant to ampicillin (75%), trimethoprim-sulphamethoxazole (50%), tetracycline (75%), chloramphenicol (62.5%), amoxicillin (62.5%) and ceftriaxone (62.5%). *E. coli* were resistant to ampicillin (100%), tetracycline (60%), and chloramphenicol (40%). *Salmonella* spp were resistant to chloramphenicol (100%), gentamycin/Ampicillin/ceftriaxone/trimethoprim-sulphamethoxazole (66.7% each). The range of resistance for Gram positive bacteria were from 23.5% – 58.8%. Fifty eight percent (58.8%) of *S. aureus* isolates were resistant to erythromycin, co-trimoxazole and penicillin-G. Relatively ciprofloxacin was fairly effective against both Gram positive and Gram negative isolates (Table 3).

**Table 3:** Resistance pattern of Gram positive and Gram negative bacteria isolated from septicemia suspected patients in Gondar University Hospital from September 1, 2004 to January 1, 2012

**Bacterial isolates**		**Antimicrobials**									
		**AMP**	**SXT**	**TTC**	**C**	**CN**	**PEN**	**E**	**CIP**	**AMX**	**CRO**
		**n**	**n**	**n**	**n**	**n**	**n**	**n**	**n**	**n**	**n**
		**(%)**	**(%)**	**(%)**	**(%)**	**(%)**	**(%)**	**(%)**	**(%)**	**(%)**	**(%)**
***Gram + ve***	*CONS*	12	16	22	12	9	15	9	7	8	11
		40	53.3	73.3	40	30	50	30	23.3	26.6	36.6
	*S. aureus*	8	10	6	4	5	10	10	5	5	6
		47	58.3	35	23.5	29.4	58.8	58.8	29.4	29.4	35.3
**Gram-ve**	*Klebsiella* spp.	6	4	6	5	3	NA	NA	2	5	5
		75	50	75	62.5	37.5			25	62.5	62.5
	*E. coli*	5	2	3	2	1	NA	NA	1	1	2
		100	40	60	40	20			20	20	40
	*P. aeroginosa*	4	3	2	3	1	NA	NA	2	2	1
		40	75	50	75	25			50	50	20
	*Salmonella* spp.	2	2	2	3	2	NA	NA	1	2	2
		66.7	66.7	66.7	100	66.7			33.3	66.7	66.7

Key:- AMX-amoxicillin, SXT-trimithoprin-sulphamethoxazole, TTC-tetracycline, C-chloramphenicol, CN-gentamycin, PEN- penicillin, E- erythromycin, CIP-ciprofloxacin, CRO-ceftriaxone , NA-Not Applicable.

## Discussion

The result of this study demonstrated that the profile of microbial isolates causing septicaemia and their susceptibility pattern to most commonly used antimicrobial agents. The rate (18.2%) of bacteria isolation in the blood culture of septicemia suspected patients in this study was in line with what had been previously reported in Nigeria (18.2%) [17]. However, this finding was relatively lower than studies done in Gondar, Ethiopia (24.2%) and Zimbabwe (37.1%) [18,19]. On the other hand, our finding was higher than a study done in Iran 4.1% [20]. The varying proportions may be due to the different methodology used and the area of study, because of the regional variation known to occur.

The range of microorganisms that invade the bloodstream has been systematically studied by several researchers. In our study, 69% of infections were caused by Gram-positive and 31% by Gram-negative bacteria. Several studies in different countries, Jimma Ethiopia, (60.9% and 39.1%) Gondar Ethiopia (70.2% and 29.8%), Zimbabwe (71.9% and 28.1%) Addis Ababa Ethiopia (62.6% and 37.4%), have shown marginally higher prevalence of Gram-positive and lower prevalence of Gram-negative organisms, respectively [12,18,19,21]. On the contrary, Gram-negative bacteria have been reported as the commonest cause of bacteremia in hospitalized febrile patients in developing countries in studies conducted in Nigeria (69.3% and 30.7%), Saudi Arabia (62.2% and 33.8%), Tanzania (69.7%, 30.3%) [17,22,23]. The possible explanation for the difference could be the difference in blood culture system, the study design, geographical location, nature of patient population, epidemiological difference of the etiological agents, and seasonal variation.

In our study, CONS, *S. aureus*, *Klebsiella* spp., *E. coli*, *Salmonella* spp., *S. pyogenes, P. aeroginosa*, *Acinetobacter* spp. and *Enterobacter* spp. were the nine most common noteworthy bacterial pathogens causing BSI. More or less similar observations have been made in cases of bacteraemia in different countries, however, the proportion and predominance of the organisms varied [5,12,18,19,22,24].

The predominant aetiological agents in our study were Gram positive organisms. It conforms with other studies [18,25]. Coagulase negative staphylococci (CONS) were the most commonly isolated bacteria and this has been also found in other studies [5,7,18,19,26]. The role of CONS in bacteraemia is divisive. Until the 1970’s, CONS were mainly recognized as a contaminant. Since then, several studies have reported increasing incidence of infections due to CONS [12,27,28].

Reports from Addis Ababa Ethiopia [21] and Zimbabwe [19] revealed that (43.3%) and (42.9%) CONS were isolated; respectively which is similar to our finding. To the contrary, this finding was higher than the study done in Jimma Ethiopia (26.1%), Gondar Ethiopia (33.3%) [12,18]. The second predominant bacteria in this study was *S. aureus* which is similar to other studies [5,7,12,18,19,26]. This finding is in contrast to study done in Tanzania, the predominant bacteria are *Salmonella* spp. followed by *E. coli*[23].

In the case of Gram negative bacteria, Klebsiella spp. (12.7%) was the predominant bacteria followed by *E. coli (7.0%)* in this study. This finding is comparable to study done in Addis Ababa Ethiopia [5] where, isolation rate of *Klebsiella* spp. and *E. coli* were (9.7%) and (8.1%); respectively. In our study, we have not isolated Haemophilus influenzae like other studies in Ethiopia [5,12,18].

In this study all cases of septicaemia, a single microorganism was isolated. This observation is in agreement with earlier reports [25,29]. However, septicaemia of polymicrobial aetiology was found in other studies [19,30]. Most clinical bacteriologists failed to report polymicrobial sepsis because of misconception of contamination, ignorance of its significance or disregard for the second organism in an already positive culture [31]. However, there is a need to correlate the occurrence of polymicrobial sepsis with clinical outcome in septicemia. A patient already infected with one microbe may have acquired the second one from the hospital environment or both the bacteria could be nosocomial in origin [32].

The result of our study showed that septicaemia was relatively higher in neonates (35.3%) which was greater than other age groups. This study has established that the disease affects all age groups but it was more noticeable in neonates than children and adults. It was observed that septicemia was most prevalent in the neonates (Table 2). This finding is supported by other studies [18,21,32].

There was a significant association between age of patient and BSI (P = 0.0004). The higher occurrence in neonatal septicemia has been reported from different parts of country [18,25]. The high occurrence of septicemia in neonates in Gondar hospital may probably be adduced to their low immune response, socio-economic status of their parents, poor hygiene practices, bottle feeding and high incidence of delivery at home. An additional effect of their low socio-economic status is exhibited by the inability of their parents to pay the hospital fees charged for delivery; consequently they deliver at home or herbalist shrines where there are no proper midwifery facilities [32].

There was no statistically significant difference in gender variation in septicemia (P = 0.526) in this study. This study showed that females were more affected than males by septicemia. Though this slight variation has been previously documented by various authors [18,32].

A study of in vitro antimicrobial susceptibility profile of the aetiological agents of septicemia has revealed that there is a growing emergence of multi-drug resistant microbes. Fifty eight percent of *S. aureus* isolated were resistant to erythromycin, trimithoprin-sulphamethoxazole (SXT) and penicillin-G which is a drug often used for initial and empirical treatment of Staphylococcal infections. About 29.4% to 58.8% of *S. aureus* isolate*s* were resistant to other commonly used antibiotics like penicillin, ampicillin, tetracycline and gentamycin. The consequences of using an ineffective drug in severe bacterial infections could be disastrous as this can complicate management and increase morbidity and mortality [13-15]. In our study, Gram negative bacteria showed highest resistance to ampicillin and chloramphenicol with resistance rate of 85.5% and 69.5%; respectively which is similar with study conducted in Iran [26].

A general overview of the antibiogram of all the bacterial isolates indicates that Gram negative bacteria exhibited a greater level of antimicrobial resistance ranging between 20%– 100%) than Gram positive bacteria (23.5% – 58.8%) to various antibacterial agents employed during the study period. This is comparable to study in Nigeria for Gram negative bacteria (19.8%-92.3% and Gram positive (10%-87%) [32]. This situation raises serious concern. This suggests a very high resistance gene pool due perhaps to gross misuse and inappropriate usage of the antibacterial agents [32].

Although the susceptibility of the organism isolated to the third generation cephalosporin was generally good in the present study, the high cost of this group of drugs precludes their use as first choice in the treatment of septicemia. Ciprofloxacin was found to be effective against both Gram positive and Gram negative isolates. Similar findings have been reported in previous studies done in Ethiopia and in India [5,12,18,21,33].

Since it is retrospective study, the study population was not systematically selected, and since a relatively low number of cultures were performed over the study time-period the results may not be truly representative. In addition it is possible that CONS isolates may in some cases represent contaminants from the skin. In addition, only aerobic cultures were performed thus limiting identification to anaerobic pathogens. Nevertheless, the data are of value with respect to antimicrobial susceptibility of BSI pathogens in Ethiopia.

## Conclusions

In the present study most of the pathogens isolated from blood showed high rate of resistance to most commonly used antibiotics used to treat bacterial infections. The study indicates the common antibiotic resistance pattern in the study area, and potentially helps in prescribing decisions avoiding the misuse of appropriate antibiotics.

## Competing interests

The authors declare that they have no competing interests.

## Authors’ contributions

MD was the primary researcher, conceived the study, designed, participated in data collection, conducted data analysis, drafted and finalized the manuscript for publication. AG, TA and TT, assisted in data collection and reviewed the initial and final drafts of the manuscript. MD, GY, AA and MG interpreted the results, and reviewed the initial and final drafts of the manuscript. All authors read and approved the final manuscript.

## References

[B1] Bailey and Scott’s Diagnostic microbiology Forbes BA, Sahm DF, Weissfeld AS A textbook for isolation and identification of pathogenic microorganisms 2002 St. Louis: The Mosby Company 378 422 In 11th edition Edited by

[B2] Diekma DJ Beekman SE Chapin KC Morel KA Munson E Deorn GV Epidemiology and outcome of nosocomial and community onset bloodstream infection J Clin Microbiol 2003 41 3655 3660 10.1128/JCM.41.8.3655-3660.2003 12904371 PMC179863

[B3] Aiker AM Mturi N Niugana P **Risk and cause of paediatrics hospital acquired bactermia Klifi district hospital, Kenya: prospective cohort study** Lancet J 2011 10 Suppl 37 2012 2017 10.1016/S0140-6736(11)61622-X PMC3242162 22133536

[B4] Daniel RK Scott AF James MB Sanjay S Brief Report: Incidence, Etiology, Risk Factors, and Outcome of Hospital acquired Fever J Gen Intern Med 2006 21 1184 1187 10.1111/j.1525-1497.2006.00566.x 17026728 PMC1831668

[B5] Asrat D Amanuel Y Prevalence and antibiotic susceptibility pattern of bacterial isolates from blood culture in Tikur Anbessa hospital, Addis Ababa Ethiopia. Ethiop Med J 2001 39 Suppl 2 97 104 11501295

[B6] James AK Mark EJ Deborah CD Clyde T Daniel FS Gregory AV Prevalence and antimicrobial susceptibilities of bacteria isolated from blood cultures of hospitalized patients in the United States in 2002 Ann Clin Microbio Antimicrobi 2004 3 Suppl 7 1 8 10.1186/1476-0711-3-7 PMC420484 15134581

[B7] Rina K Nadeem SR Kee PN Parasakthi N Etiology of blood culture isolates among patients in a multidisciplinary teaching hospital in Kuala Lumpur J Microbiol Immunol Infect 2007 40 432 437 17932604

[B8] Manjula M Pyria D Varsha G Antimicrobial susceptibility pattern of blood isolates from a teaching Hospital in north India Japan J Infec Dis 2005 58 174 176 15973011

[B9] Becker JU Theodosis C Jacob ST Wira CR Groce NE Surviving sepsis in low-income and middle-income countries: new directions for care and research Lancet Infect Dis 2009 9 Suppl 9 577 582 19695494 10.1016/S1473-3099(09)70135-5

[B10] Tziamabos AO Kasper DL Principle and practice of infectious diseases Frank Polizano J 2005 26 2810 2816

[B11] Madsen KH Sorensen HT **Secular trends in incidence and mortality of bacteremia in Danish country** APMIS 1999 107 346 352 10.1111/j.1699-0463.1999.tb01563.x 10223308

[B12] Zenebe T Kannan S Yilma D Beyene G Invasive Bacterial Pathogens and their antibiotic susceptibility patterns in Jimma University specialized Hospital, Jimma, South West Ethiopia Ethiop J Health Sci 20011 21 Suppl 1 1 8 22434980 10.4314/ejhs.v21i1.69038 PMC3275852

[B13] Harbarth S Ferrière K Hugonnet S Ricou B Suter P Pittet D Epidemiology and prognostic determinants of bloodstream infections in surgical intensive care Arch Surg 2002 137 1353 1359 10.1001/archsurg.137.12.1353 12470098

[B14] Ibrahim EH Sherman G Ward S The influence of inadequate antimicrobial treatment of bloodstream infections on patient outcomes in the ICU setting Chest 2000 118 146 155 10.1378/chest.118.1.146 10893372

[B15] Behrendt G Schneider S Brodt HR **Influence of antimicrobial treatment on mortality in septicemia** J Chemo-therap 1999 11 179 186 10.1179/joc.1999.11.3.179 10435678

[B16] Wayne PA Performance standards of antimicrobial susceptibility. National Committee for Clinical Laboratory Standards (NCCLS). NCCLS approved standards 2002 M 100 M 159

[B17] Nwadioha I Nwokedi EOP Kashibu E Odimayo MS Okwori EE A review of bacterial isolates in blood cultures of children with suspected septicemia in a Nigerian African J Microbiol Res 2010 4 Suppl 4 222 225

[B18] Ali J Kebede Y Frequency of isolation and antimicrobial susceptibility pattern of bacterial isolation from blood culture in Gondar University Hospital Ethio Med J 2008 46 2 155 161 21309205

[B19] Obi CL Mazarura E Aerobic bacteria isolated from blood cultures of patients and their antibiotic susceptibilities in Harare, Zimbabwe Cent Afr J Med 1996 42 Suppl 12 332 336 9164012

[B20] Seyyed MH Identification of bacteriological agents and antimicrobial susceptibility of neonatal sepsis Afr J microbial 2011 5 Suppl 5 528 531

[B21] Shitaye D Asrat D Woldeamanuel Y Worku B Risk factors and etiology of neonatal sepsis in Tikur Anbessa University Hospital, Ethiopia. Ethiop Med J 2010 48 Suppl 1 11 21 20607993

[B22] Elbashier AM Malik AG Knot AP Blood stream infections: micro-organisms, risk factors and mortality rate in Qatif Central Hospital Ann Saudi Med 1998 18 Suppl 2 176 180 17341956 10.5144/0256-4947.1998.176

[B23] Meremo A Mshana SE Kidenya BR Kabangila R Peck R Kataraihya JB High prevalence of Non–typhoid salmonella bacteraemia among febrile HIV adult patients admitted at a tertiary Hospital, North-Western Tanzania Int Arch Med 2012 5 28 10.1186/1755-7682-5-28 23075077 PMC3540015

[B24] Wisplinghoff H Bischoff T Tallent SM Seifert H Wenzel RP Edmond MB Nosocomial bloodstream infections in US hospitals: analysis of 24,179 cases from a prospective nationwide surveillance study Clin Infect Dis 2004 39 Suppl 3 309 317 15306996 10.1086/421946

[B25] Angyo IA Opkeh ES Opajobi SO Predominant bacterial agents of childhood septicaemia in Jos Niger J Med 2001 10 75 77 11705063

[B26] Rahbar M Gra-Agaji R Hashemi S Nosocomial blood stream infections in Imam Khomeini Hospital, Urmia, Islamic Republic of Iran, 1999-2001 East Mediterr Health J 2005 11 Suppl 3 478 484 16602469

[B27] Boisson K Thouverez M Talon D Bertrand X Characterization of coagulase-negative staphylococci isolated from blood infections: incidence, susceptibility to glycopeptides, and molecular epidemiology Eur J Clin Microbiol Infect Dis 2002 21 Suppl 9 660 665 12373498 10.1007/s10096-002-0799-9

[B28] Marshall SA Wilke WW Pfaller MA Jones RN *Staphylococcus aureus* and coagulase-negative staphylococci from blood stream infections: frequency of occurrence, antimicrobial susceptibility, and molecular (mecA) characterization of oxacillin resistance in the SCOPE program Diagn Microbiol Infect Dis 1998 30 Suppl 3 205 214 9572028 10.1016/s0732-8893(97)00212-5

[B29] Ghanshyam DK Ramachandram VC Piyush G Bacteriological analysis of blood culture Malaysian J Microbio 2008 4 Suppl 2 51 61

[B30] Ghanshyam DK Ramachandram VC Piyush G Bacteriological analysis of blood culture isolates from neonates in a tertiary care hospital in India J Health Popul Nutr 2002 20 Suppl 4 343 347 12659415

[B31] Mathur M Shah H Dixit K Khambdkone S Chakrapani A Irani S Bacteriological profile of neonatal septicaemia cases (for the years 1990-91) J Postgraduate Med 1994 40 18 20 8568708

[B32] Komolafe AO Adegoke AA Incidence of bacterial Septicaemia in Ile-Ife Metropolis, Nigeria Malaysian J Microbio 2008 4 Suppl 2 51 61

[B33] Kapoor L Randhawa VS Deb M Microbiological profile of neonatal septicemia in a pediatric care hospital in Delhi J Commun Dis 2005 37 227 232 17080707

